# Search interest in alleged COVID-19 treatments over the pandemic period: the impact of mass news media

**DOI:** 10.1101/2024.11.20.24317650

**Published:** 2024-11-22

**Authors:** Emily E. Ricotta, Samantha Bents, Brendan Lawler, Brianna A. Smith, Maimuna S. Majumder

**Affiliations:** 1. Department of Preventive Medicine and Biostatistics, Uniformed Services University of the Health Sciences, 4301 Jones Bridge Road, Bethesda, MD, USA 20814; 2. Division of Intramural Research (DIR), National Institute of Allergy and Infectious Diseases (NIAID), National Institutes of Health (NIH), 9000 Rockville Pike, Bethesda, MD, USA 20892; 3. CompEpi Dispersed Volunteer Research Network, 401 Park Drive, Boston, MA, USA; 4. Fogarty International Center, NIH, 9000 Rockville Pike, Bethesda, MD, USA; 5. Epidemiology and Population Studies Unit, DIR, NIAID, NIH, 5601 Fishers Lane, Bethesda, Maryland, USA 20852; 6. Boston Children’s Hospital, Computational Health Informatics Program, 300 Longwood Avenue, Boston, MA, USA; 7. Political Science, United States Naval Academy, 121 Blake Rd, Annapolis, MD, USA 21402; 8. Harvard Medical School, Department of Pediatrics, 25 Shattuck Street, Boston, MA, USA

## Abstract

**Background:**

Understanding how individuals obtain medical information, especially amid changing guidance, is important for improving outreach and communication strategies. In particular, during a public health emergency, interest in unsafe or illegitimate medications can delay access to appropriate treatments and foster mistrust in the medical system, which can be detrimental at both individual and population levels. It is thus key to understand factors associated with said interest.

**Methods:**

We obtained US-based Google Search Trends and Media Cloud data from 2019–2022 to assess the relationship between Internet search interest and media coverage in three purported COVID-19 treatments: hydroxychloroquine, ivermectin, and remdesivir. We first conducted anomaly detection in the treatment-specific search interest data to detect periods of interest above pre-pandemic baseline; we then used multilevel negative binomial regression—controlling for political leaning, rurality, and social vulnerability—to test for associations between treatment-specific search interest and media coverage.

**Findings:**

We observed that interest in hydroxychloroquine and remdesivir peaked early in 2020 and then subsided, while peak interest in ivermectin occurred later but was more sustained. We detected significant associations between media coverage and search interest for all three treatments. The strongest association was observed for ivermectin, in which a single standard deviation increase in media coverage was associated with more than double the search interest (164%, 95% CI: 148, 180), compared to a 109% increase (95% CI: 101, 118) for hydroxychloroquine and a 49% increase (95% CI: 43, 55) for remdesivir.

**Interpretation:**

Search interest in purported COVID-19 treatments was significantly associated with contemporaneous media coverage, with the highest impact on interest in ivermectin, a treatment demonstrated to be ineffectual for treating COVID-19 and potentially dangerous if used inappropriately.

**Funding:**

This work was funded in part by the US National Institutes of Health and the US National Science Foundation.

## Introduction

Throughout the early COVID-19 pandemic, several repurposed drugs were evaluated as potential COVID-19 treatments in the United States, including hydroxychloroquine, ivermectin, and remdesivir. Hydroxychloroquine, an antimalarial and anti-rheumatic drug, gained US Food and Drug Administration (FDA) emergency use authorization to treat severe COVID-19 hospitalizations in March 2020; this was shortly revoked after clinical trials showed harmful cardiovascular side effects at the doses being dispensed.^1^ Ivermectin, an antihelminthic, was proposed as an antiviral agent for COVID-19 in early 2020, leading to the initiation of several randomized control trials between mid-2020 and 2021;^2^ however, results were generally inconsistent and inconclusive, leading the FDA, US National Institutes of Health, and World Health Organization to recommend against its use in 2021.^3^ Remdesivir, an antiviral originally developed to treat hepatitis C, was approved by the FDA for persons hospitalized with COVID-19 in October 2020 after multiple clinical trials showed moderately improved outcomes among hospitalized cases.^4^ Public interest in these treatments varied over time, driven partially by governmental, political, and media response to current events.^5^ Indeed, for hydroxychloroquine and then ivermectin, public demand for the drugs caused widespread pharmacy shortages, and in the case of ivermectin—a drug commonly used for livestock deworming—shortages of veterinary ivermectin as well.^6,7^

Beyond causing shortages, public interest in unsafe or illegitimate medications during a public health emergency can delay access to appropriate treatments and foster mistrust in the medical system, a detriment at both individual and population levels. Therefore, both for situational awareness and interventional purposes, it is critical for medical and policy communities to understand how individuals obtain medical information during an emerging crisis.

One method of evaluating public interest in medical information is the use of Internet search data. These data can serve as a large-scale, near real-time proxy for public interest by providing insight into what information people seek online including the subject, timing, and frequency of specific topics. A recent study in Switzerland found that 55% of Google internet searches were informational (as opposed to transactional or navigational),^8^ and in 2019, roughly 7% of Google searches (about 1 billion searches daily) were health related.^9^ This makes Internet search data—publicly available through Google Search Trends (GST)—an attractive tool for health information research;^10^ indeed, GST was used extensively throughout the pandemic to measure population engagement with public health messaging on topics like masking, vaccines, and misinformation, as well as interest in COVID-19 more broadly.^11,12^

Another way to gauge public interest in a topic is by assessing relevant news media coverage—a data source that is particularly useful for topics like uncommon medications, which would rarely enter the public zeitgeist otherwise. The impact of news media on public interest and changing health behaviors is well-studied,^13,14^ including during the COVID-19 era.^11,15,16^ Previous research by our team and others have assessed a variety of health-related topics and their relationship with news media coverage using services like Media Cloud (MC),^17^ an open-source content analysis tool that collects time series of national and sub-national news media coverage, comprising data from more than 50,000 news sources since its inception. The bulk of MC’s corpora are news stories from web-based media sites, which is particularly relevant as 86% of US adults reported getting news from an online source in 2023.^18^

Interestingly, the relationship between public interest and media coverage is not unidirectional; studies on communication theory demonstrate that the media can also serve as a reflection of public opinion rather than function solely as its driver–a phenomenon known as the “reflection hypothesis”.^19^ As interest in a topic increases, people begin searching for things they see on the news, while in response, the news covers things they know people are interested in. This study aimed to investigate the bidirectional relationship between online search interest and media coverage as a way to evaluate exposure to and access of health-related information during the COVID-19 pandemic by evaluating: 1) the extent to which US news sources covered supposed COVID-19 treatments, 2) the extent of public interest in these treatments, as reflected by online search interest, and, 3) the relationship between these data sources within the US.

## Methods

### Data sources for exposure and outcome

We used two primary data sources to assess the relationship between treatment-related news media coverage (exposure) and treatment-related search interest (outcome) in the US: MC and GST. This study was not intended to assess a causal relationship between these two data sources; MC as exposure and GST as outcome are arbitrary definitions assigned to a truly bidirectional relationship for statistical purposes. All data were obtained for the period from January 2019 through October 2022 using the queries “hydroxychloroquine”, “ivermectin”, and “remdesivir” for both MC and GST. Data from 2019 were included in the analysis to provide a pre-pandemic baseline for each of these search terms; data collection ceased in 2022 after a sustained period of low search interest and media coverage of all three treatments.

Daily treatment-related news media coverage data were downloaded from MC’s state-level news corpora. These corpora include media articles published in state-specific news sources (e.g., *Arkansas Times*) and report daily normalized content percentage, i.e., the number of corpus-specific articles reporting on a topic of interest on a given day divided by the total number of corpus-specific articles on the same day. Using corpora for each US state, daily content percentages were aggregated into average weekly time series for each treatment.

Meanwhile, weekly, state-level time series of treatment-specific Google search interest were collected via the *gtrends* R package. Notably, search interest data are normalized by Google; the number of topic-specific searches is divided by the total searches conducted within a specified geography and time period, making GST data both fractional and relative to the region and time window inputted. The data are scaled from 0–100, with a score of 100 representing peak search interest in the selected geography (i.e., state) and time interval. We obtained national-level GST for anomaly detection and state-level GST for regression analysis, described below.

### National-level anomaly detection in treatment-specific search interest

To identify peaks in treatment-related search interest, we performed anomaly detection for each treatment on weekly search interest time series at the national level using the *Anomalize* package in R, with alpha set at 0.05. This package uses the median and interquartile range (IQR) of GST to establish baseline, applies a modifier based on alpha, and then determines whether the level of search interest exceeds that threshold. In our data, an anomaly was observed if GST was >5 for hydroxychloroquine, >10 for ivermectin, and >11 for remdesivir. For each detected anomaly, we also searched the Internet for potentially relevant current events that could have resulted in increased media attention or public awareness.

### State-level association of treatment-specific search interest and media coverage

To visualize geographic heterogeneity in search interest and media coverage, we created heatmaps at the state level for each treatment using *ggplot2*. Then, using treatment-specific regression models, we assessed the state-level relationship between treatment-specific media coverage and contemporaneous (i.e., non-lagged) search interest. We considered three covariates in the models: political leaning, rurality, and social vulnerability.

As COVID-19 treatments were highly politicized during the initial stages of the pandemic,^9^ we hypothesized that statewide political leaning may have differentially influenced interest in specific treatments. We measured statewide political leaning as the percentage of a given state that voted for the Republican Party candidate in the 2020 presidential election, on a 0–100 scale.^20^

Social vulnerability may have also played a role in treatment interest given its association with reduced access to healthcare services.^21^ Reduced interaction with healthcare providers and services has previously been associated with riskier health choices and poor health outcomes, providing evidence that social vulnerability may influence treatment-seeking behavior.^21,22^ We measured social vulnerability using the COVID-19 Community Vulnerability Index (CCVI), which provides a state-level measure of access to healthcare, taking economic, structural, and demographic barriers into account.^23^ State-level scores are scaled continuously from 0–100, where 100 represents the highest level of vulnerability.

Lastly, we included rurality in the models, first as a proxy for known barriers to healthcare that could potentially influence off-label medication use,^24^ and second because of ivermectin’s widespread use on farms as a livestock deworming agent, which we hypothesized could influence search interest especially among farming communities. Rurality was measured using the 2021 US Census Bureau Urban and Rural dataset, which provides the percentage of a given state’s population that lives in rural settings, measured on a 0–100 scale, with 100 being the most rural.^25^

Fitting zero-inflated negative binomial models to account for overdispersion in weekly state-level search interest, we regressed search interest against weekly media coverage for each treatment using the *glmmTMB* package in R. All covariates were scaled and mean-centered, and we included “state” as a random intercept to control for interstate variability in the impact of covariates on the exposure (i.e., media coverage). To compare effect sizes between treatments, we included an interaction term between treatment and media coverage; ivermectin served as the reference group. Slopes were compared and plotted using the *emmeans* R package by calculating the estimated marginal means of the linear trends. P-values were adjusted using the Tukey method. Model results are presented as percent difference in search interest with 95% Wald confidence intervals. All analyses were performed using R version 4.3.x.^26^

## Results

**Figure 1.**
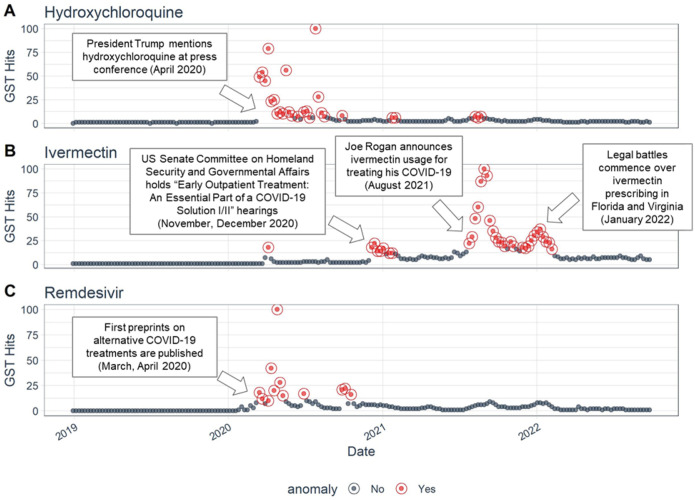
National GST time series for a) hydroxychloroquine, b) ivermectin, and c) remdesivir from 2019–2022. Identified anomalies are shown in red and non-anomaly time points are shown in gray. GST = Google Search Trends. Annotated with COVID-19 treatment-related events occurring contemporaneously with the GST anomalies.

Nationally, the average search interest (out of 100) was 2·5 for hydroxychloroquine (standard deviation [SD]: 8·7), 7·0 for ivermectin (SD: 11·6), and 2.8 for remdesivir (SD: 8·3) during the pandemic period (January 2020-October 2022), compared to 0·5 (SD: 2·0), 1.4 (SD: 3·8), and 0·5 (SD: 3·6) for each treatment, respectively, in 2019. Nationally averaged media coverage was 0·08% for hydroxychloroquine (SD: 0·3), 0·02% for ivermectin (SD: 0·16), and 0·08% for remdesivir (SD: 0·35) over the same time interval, compared to ≤0·0006% (SD<0·02) for each of the treatments in 2019. Using anomaly detection to identify periods of high interest (Fig 1.), we observed that search interest in hydroxychloroquine and remdesivir peaked early in 2020 and subsided by September and October 2020, respectively. While no further anomalies were detected for remdesivir, two additional small anomalies were detected for hydroxychloroquine in February and August 2021. Interest in ivermectin began as early as December 2020 and was sustained through February 2022, with peak interest occurring in late August 2021.

**Figure 2.**
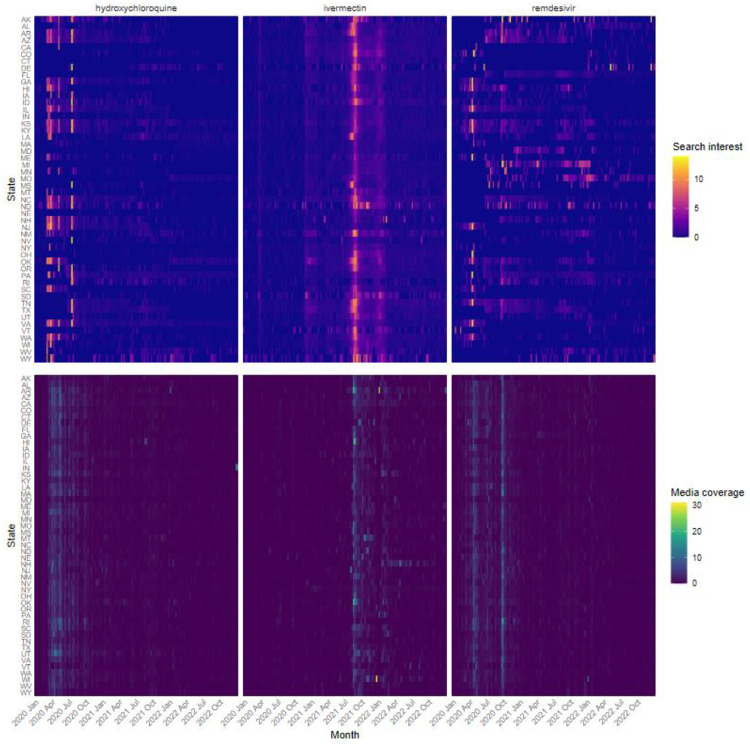
Search interest and media coverage for each state over the study period for each of the three treatments.

**Figure 3.**
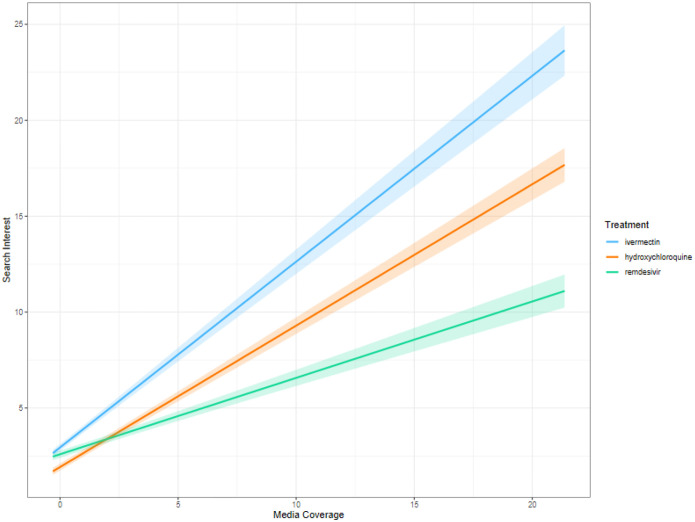
Regression slopes for the relationship between treatment-specific search interest and media coverage. Slopes were calculated using zero-inflated negative binomial regression, controlling for state-level rurality, political leaning, and social vulnerability. To compare the difference in magnitude between the treatments, we calculated the estimated marginal means. All trends were significantly different (p<0.001).

To further investigate patterns in search interest and media coverage, we next evaluated data at the state level. Heatmaps of weekly search interest and media coverage for each state allowed us to observe heterogeneity across the country in both measures (Fig. 2). When controlling for average state political leaning, rurality, and social vulnerability, we found significant differences in the level of search interest for each treatment as well as a significant association between media coverage and contemporaneous search interest for all three treatments. Compared to ivermectin, search interest was 64% lower (95% CI: 62, 66) for hydroxychloroquine and 30% lower (95% CI: 25, 35) for remdesivir, holding media coverage constant. When evaluating the impact of media coverage on search interest, the strongest association was observed for ivermectin, where a single standard deviation increase in media coverage was associated with more than double the search interest (164%, 95% CI: 148, 180), compared to a 109% increase (95% CI: 101, 118) for hydroxychloroquine and a 49% increase (95% CI: 43, 55) for remdesivir. Interestingly, while search interest was on average higher for remdesivir than hydroxychloroquine, media coverage had a more pronounced impact on search interest for hydroxychloroquine — a difference in slope of 40% (p<0.001) (Fig. 3).

## Discussion

In this study we identified significant public interest in three purported COVID-19 treatments (i.e., hydroxychloroquine, ivermectin, and remdesivir), as represented by anomalies detected in GST and heightened search interest in these medications during the pandemic period. Additionally, we found significant associations between news media coverage and all three treatments. This association was significantly larger for ivermectin, an incredibly popular but illegitimate treatment for COVID-19, compared to remdesivir, the only treatment which remains FDA-approved for use,^3,4^ and hydroxychloroquine, an initially FDA-authorized treatment with early potential that ultimately proved ineffective and dangerous.^1^ That the strength of relationship between ivermectin search interest and media coverage was highest despite ivermectin receiving the lowest news coverage warrants further examination. One explanation could be that people were receiving information about ivermectin from non-news media sources such as contact networks, social media, podcasts, or elsewhere. As these platforms are increasingly important for communication in general, it is important to consider their role in the dissemination of health information. Another hypothesis explaining the heightened interest in ivermectin specifically is that media coverage of ivermectin had a stronger impact on search behavior than did articles about the other treatments. For example, if articles about ivermectin were more sensational and/or incendiary, that may explain the increased interest in ivermectin (despite less coverage). Future studies should assess the content and sentiment of health-related news articles to understand the kind of language that is most likely to prompt heightened search interest so we can leverage those techniques for beneficial health communication strategies.

At the beginning of the pandemic, there was considerable uncertainty surrounding the nature of COVID-19, particularly effective medications and treatments. As the scientific community conducted more studies and amassed new results, public health guidance changed to reflect updated knowledge.^27^ Although this is to be expected in the earliest days of a novel pandemic, a lack of consistent and transparent messaging from public health officials contributed to the general public relying more heavily on non-expert information sources like politicians, public figures, and celebrities for public health guidance.^5^ In addition, recent studies report that the percentage of Americans who regularly see a primary care physician has steadily decreased between 2014 and 2022, meaning regular communication with licensed medical providers is becoming more limited for many communities across the US.^28^ These factors may have contributed to the general public’s increasing reliance on other information sources—such as news media—for guidance during the COVID-19 pandemic. Indeed, over the last several years perceived US media influence on government decisions has grown among the public and will likely continue to have an outsized influence on public interest.^29^

We note several limitations in this analysis. First, the news media corpora we assessed only includes state-level media outlets, as MC is not able to track state-level readership of national news sources. Given that many individuals rely heavily on national media outlets for news, the media exposure used in this analysis may be skewed if individuals within a state engage differently with national- versus state-level news outlets. Additionally, larger states had relatively larger news media corpora than smaller states; however, no corpus reported fewer than 30 sources, indicating that sample sizes were sufficient to assess news media coverage at this geographic scale. Notably, MC does not capture sentiment (i.e., the emotions and attitudes portrayed by news media about a particular issue), meaning we were unable to comment on whether the treatment-related news media sources were reporting positively or negatively on purported treatments. Sentiment analysis of media coverage relating to COVID-19 treatments is an area of interest for future research. Because data were aggregated by week (due to our inability to acquire daily GST over a large temporal period), we could not use methods such as temporal lagging to assess a causal relationship between MC and GST. However, we also do not theorize a straightforward unidirectional relationship between these variables—search interest and media coverage likely impact each other near simultaneously. Lastly, public interest was only captured for the population using Google as their primary search engine; people using alternate engines like Bing or DuckDuckGo were not captured in our study. However, in the US, the Google search engine averages nearly 90% of the market share;^30^ we are therefore confident that our results capture most search interest.

## Conclusions

Regardless of the quality of public health messaging available to the general public, social media, news outlets, and political and public figures will continue to be a major influence on how individuals seek health-related guidance. During a public health emergency, the information that populations access can directly influence health-seeking behaviors, with potentially life-threatening consequences. More broadly, positive media coverage of unsafe or unapproved medications can deter individuals from trusting and accessing safe alternatives that are more likely to be efficacious in preventing disease progression. Given the strong association between treatment-related news media coverage and public interest in said treatments, our results suggest that news media has potential as a mechanism for experts to understand the landscape of public opinion and to reach audiences during future public health emergencies.

## Data Availability

All data used in this study are available through public databases.

## Research in context

### Evidence before this study:

We reviewed the existing literature on public interest in COVID-19 treatments during the pandemic, with a focus on studies using internet search data. Databases included PubMed, Google Scholar, and Embase. For example, in Embase, we iterated on search terms such as (‘SARS-CoV-2’ OR ‘nCoV’ OR ‘COVID-19’) AND (‘treatment’/exp OR ‘treatment’ OR ‘medicine’/exp OR ‘medicine’) combined with (‘search engine’/exp OR ‘search engine’ OR ‘google’/exp OR ‘google’ OR ‘google search trends’) for GST; or (‘publication’/exp OR ‘publication’ OR ‘news’/exp OR ‘news’) AND (‘behavior’/exp OR ‘behavior’ OR ‘sentiment analysis’/exp OR ‘sentiment analysis’ OR ‘public opinion’/exp OR ‘public opinion’) to find relevant articles. Searches were not restricted to the English language. Several studies were found that used internet search data or discussed how news media impacts public opinion related to health topics. These methods were uncommon in the literature, and none were found to use these data sources to examine public opinion about potential COVID-19 treatments.

### Added value of this study:

This study adds to the existing evidence by specifically examining the relationship between public interest in and news media coverage about three specific purported COVID-19 treatments: hydroxychloroquine, ivermectin, and remdesivir. It uses a robust methodological approach, combining internet search interest data and news media coverage with state-level covariates like political leaning, rurality, and social vulnerability. This provides a nuanced understanding of how media coverage and sociopolitical factors interact to influence public interest in clinically appropriate, potentially controversial, or unproven medical treatments.

### Implications of all the available evidence:

The findings highlight the significant influence of news media coverage on public interest in medical treatments, particularly during a public health crisis. This emphasizes the need for responsible reporting and the potential for utilizing media as a tool for effective health communication. However, the study also reveals the susceptibility of the public to misinformation and the potential for heightened interest in treatments lacking scientific evidence. This underscores the importance of critical media literacy and the need for clear and consistent communication from public health officials to counteract the spread of misinformation. Future research should focus on understanding the mechanisms driving the relationship between media coverage and public interest, including the role of social media and other information sources.
